# Sex-specific effects of birth weight on longitudinal behavioural outcomes in children and adolescents: findings from the raine study

**DOI:** 10.1007/s00787-024-02450-6

**Published:** 2024-05-09

**Authors:** Lars Meinertz Byg, Carol Wang, John Attia, Craig Pennell

**Affiliations:** 1 School of Medicine and Public Health, University of Newcastle, Newcastle, NSW Australia; 2 Mothers and Babies Research Centre, Hunter Medical Research Institute, Lot 1 Kookaburra Cct, New Lambton Heights NSW 2305, 04 23215758, Newcastle, NSW Australia; 3 Department of Medicine, John Hunter Hospital, Newcastle, NSW Australia

**Keywords:** Birth weight, Sex differences, Child Behaviour Checklist, Attention Problems, Social Problems, Aggression Problems, Longitudinal

## Abstract

Previous cross-sectional studies suggest that birth weight (BW) is associated with aggression-, social- and attention problems differently in boys and girls. We sought to test if these differences could be confirmed in a longitudinal study. The 1989 Raine Study provided prospectively collected data on perinatal variables and repeated child behaviour checklist assessments from ages 5 to 17. Linear mixed effects models provided crude and adjusted relationships between BW and childhood behaviour at a conservative significance threshold using prenatal maternal covariables in adjusted models. Sensitivity analyses included an age10 teacher assessment. Data on behaviour, BW and sex, was available in 2269 participants. Male sex was associated with increased aggression problems at lower BW compared to females in the crude model (Interaction B: -0.436, 98.3%CI: [-0.844, -0.0253]), but not the adjusted model (Interaction B: -0.310, 98.3%CI: [-0.742, 0.140]). Male sex was associated with increased attention problems at lower BW compared to females in both the crude model (Interaction B: -0.334, 98.3%CI: [-0.530, -0.137]) and the adjusted model (Interaction B: -0.274, 98.3%CI: [-0.507, -0.0432]). Male sex was associated with increased social problems at lower BW compared to females in both the crude model (Interaction B: -0.164, 98.3%CI: [-0.283, -0.0441]) and the adjusted model (Interaction B: -0.148, 98.3%CI: [-0.285, -0.00734]). Using repeated measures from ages 5–17 we were able to show a crude and adjusted male vulnerability to lower BW in the development of attention problems and social problems. We did not find a BW x sex interaction for the development of aggressive behaviour.

**Supplementary Information:**

The online version contains supplementary material available at 10.1007/s00787-024-02450-6.

## Introduction

Pre- and perinatal events are associated with childhood and adult behaviour and mental illness [1]. Low birth weight (LBW) is a well-studied risk factor in the field of developmental origin of health and disease (DOHaD) [2] and predicts adult psychopathology and violent criminal behaviour [3–6]. Similarly, childhood behaviour has been associated with LBW in multiple settings, including twin studies [7–11]. There are two primary reasons why babies have LBW: they are either constitutionally small with symmetric fetal growth, or they have fetal growth restriction, which is associated with more neurodevelopmental deficits [12]. The latter is typically marked by asymmetric fetal growth (larger head than abdomen) as blood/nutrient flow is diverted to the developing brain. Male fetal sex in LBW is associated with more asymmetric growth [13], more perinatal complications [14] and possibly more early neurodevelopmental deficits [15–18], suggesting male vulnerability. Given widespread sex dimorphism in normal fetal neurodevelopment [19], neuropsychiatric behavioural correlates of LBW could similarly differ, but reports of behavioural sex differences from LBW in childhood and adolescence have been inconsistent [20–23].

Murray et al., who examined the childhood behaviour checklist (CBCL) attention problems [24, 25], found stronger effects of reduced fetal growth in females compared to males [21]. In this study, 3700 individuals from the Brazilian PELOTAS birth cohort were followed prospectively with confounders recorded during pregnancy and behavioural assessment at age four. In contrast, Momany et al., using the DSM-IV ADHD Rating Scale and the Conners’ Rating Scale–Revised Short Form, found that males with lower BW had a stronger association with externalising and ADHD behaviour at age 12 [22] in 900 individuals from North America. In this study, parents recalled confounders and BW, giving rise to the risk of recall bias. A meta-analysis from 2018 by the same author did not find an influence of sex on effect size of BW on ADHD but demonstrated that use of categories for both BW and ADHD measures resulted in significant heterogeneity in effect sizes [26]. Interestingly, a high-powered study by Dooley et al. subsequently examined the sex-specific effects of continuous BW on all continuous CBCL-scores [25]. In this paper, nearly 10 000 individuals in the ABCD cohort were assessed at age 9–11 with subsequent testing of the association between BW and CBCL subscales- and total score [20]. At a conservative significance threshold, Dooley et al. found an inverse association between BW and total CBCL scores, attention problems and aggression problems driven by males [20]; additionally, social problems had a nominally significant increase in males with lower BW compared to females, but the sex interaction was insignificant after correction for multiple testing. A limitation of the ABCD study is the reliance on parental recollection of BW and potential confounders and the single behaviour assessment. The cross-sectional nature of previous studies limits conclusions regarding persistent sex differences as low BW and biological sex are also implicated as determinants of behavioural phenotype trajectories across childhood and adolescence [27, 28]. A male vulnerability to attention- and peer problems from lower BW was partly supported by a recent study using repeated measures of the strength and difficulties questionnaire from ages 9–17 but the interaction was not significant. In addition, the study’s information on familial confounders of BW was collected from age 9 onward and could represent downstream consequences of childhood behaviour [23]. The conflicting results at different ages and limitations cited above leave the question of a persistent sex difference unresolved.

This paper aims to test sex differences in the relationship between BW and outcomes of aggression-, social- and attention problems. We seek to add to previous knowledge by using repeated measures across childhood and adolescence and to adjust for confounders collected during pregnancy to avoid recall bias.

## Participants and methods

### Study sample

We analysed data from the Raine Study (https://rainestudy.org.au/) [29]. The Raine Study is a longitudinal study following mother-baby dyads recruited at or around 18 weeks gestation (*n* = 2979) through the public antenatal clinic at King Edward Memorial Hospital and nearby private clinics in Perth, Western Australia, from May 1989 to November 1991. Offspring with information on BW and sex were followed up throughout childhood for behavioural assessments (age 5 *n* = 2058, age 8 *n* = 1978, age 10 = 1915, age 14 *n* = 1697, age 17 *n* = 1314) with attrition of mothers with lower age, education, income and non-European ancestry [30, 31]. The Human Research Ethics Committees at the University of Western Australia, King Edward Memorial Hospital, and Princess Margaret Hospital in Perth, Australia, granted ethics approval for each follow-up in the study.

### Outcome variables

Using the Achenbach System of Empirical Assessment (ASEBA) CBCL for Ages 4–18 (CBCL/4–18), we derived scores for attention problems, aggressive behaviour and social problems at ages 5, 8, 10, 14 and 17 based on the report by Dooley et al. [20]. The CBCL/4–18 is a commonly used dimensional measure of child behaviour during the previous six months. The complete questionnaire contains 118 items and shows good internal reliability and validity in several population settings [24]. Participants were excluded from the analysis if they were missing more than 8 items on the entire CBCL [32]. The attention problem subscale measures both problems of attention, impulsivity and hyperactivity and consists of 11 items (score 0–22). The social problems scale measures peer interaction problems and consists of 8 items (score 0–16). The aggressive behaviour scale consists of 20 items (score 0–40). The CBCL is a highly validated psychometric tool and is used in the clinical setting as a guide and screening tool [24, 25, 33], with reproducibility of an 8-factor structure across countries [34]. Previous authors have used the CBCL raw scores (and not T-score corrected for age and sex) to examine sex differences [20, 21]. We chose the same approach of regressing the raw scores. The CBCL syndrome scores across populations tend to be right skewed and the clinical cut-offs for the raw scores are therefore low (for attention problems clinical relevance is assumed around 7 to 8 points depending on sex and age). Post-hoc we used T-scores to derive a clinical “borderline” score in each of the 3 domains (T-score cut-off > = 67).

We used two additional instruments for sensitivity analyses: the 1991 ASEBA preschool form of the CBCL (also filled out by parents) and the Teacher Report Form (TRF). The preschool CBCL scale for aggressive behaviour was assessed at two years of age and had 33 items (score 0–66). The 1991 edition of the preschool questionnaire did not have a validated attention problem and social problem scale. The TRF is another well-validated form under ASEBA and can be used in the clinical setting to support findings from the CBCL [35]. The TRF attention problems scale has 20 items (score 0–40), the aggression problems scale has 25 items (score 0–50) and the social problems scale has 13 items (score 0–26).

### Early life determinants and potential confounders

Gestational age (GA) in weeks was determined either by the date of the last menstrual period (LMP) or fetal biometry at the 18-week gestation ultrasound (USS) examination. Maternal age, BW and fetal sex were retrieved from hospital records. Different populations have different normal spectra for BW [36], and we used the continuous, normalised BW as the exposure by subtracting our sample BW mean and dividing by the sample standard deviation. Post hoc, a dichotomized variable corresponding to a LBW was derived (< 2500 g cut-off).

Information on confounders was recorded at prenatal visits at gestational week 18 by maternal questionnaire. As pregnancy-related risks are increased in mothers with predisposition for mental illness [37] we included factors that could be associated with both BW and behavioural outcomes. The included factors were maternal education, maternal psychiatric illness, maternal smoking during pregnancy, maternal diabetes mellitus or hypertension, family income, maternal ethnicity and maternal alcohol consumption (for questionnaire formulation see supplementary materials). No post-natal variables were used, to avoid adjusting for downstream consequences of BW. We did, however, include the cohort age at assessment as a fixed term to minimise the noise from age-related CBCL-score reductions. Potential confounders were inserted in a directed acyclic graph (supplementary Fig. 1) to help us decide on the co-variables to include in our models. Smoking, alcohol consumption, economic class and maternal education were recorded as ordinal 5–6 level variables and were treated as continuous in the multivariable analysis.

### Statistics

All statistical analyses and graphs were performed in “R” [38] and its associated libraries “Gmisc”, “lme4”, “lmeresampler”, “lmertest” and “boot”. The Wilcoxon rank-sum test was used to compare sample distributions in the continuous baseline variables and CBCL outcomes between males and females.

Testing the associations between BW and behavioural outcomes was done with mixed-effects modelling to avoid pseudo-replication from repeated measurements of the same participant. Recent work has demonstrated that the treatment of ordinal data as continuous does not impact inference in most situations [39], and linear mixed modelling is robust to missing data and violation of distributional assumptions [40]. We, therefore, chose to treat the CBCL scores as continuous variables. As we examined three different (albeit correlated) outcomes, we applied a Bonferroni-correction of 3 for our alpha, meaning statistical significance was set at $\frac{0.05}{3}=0.01667$.

Model diagnostics were evaluated by examining histograms and qq-plots of residuals and random effects. CBCL subscale scores are highly right-skewed, and our residuals and random-effects displayed significant violations of distributional assumption. We, therefore, performed a non-parametric bootstrap at the participant-ID level with 5000 simulations, as suggested by Thai et al. [41], to derive estimates and 98.3% confidence intervals, which were then used for primary inference. Approximate *p*-values were calculated from the bootstrapped estimate z-statistic. For the sensitivity analysis using the TRF (see below) we performed a simple linear regression of TRF-scores as described below, and subsequently did a non-parametric bootstrap with 5000 simulations because of non-normality in our residuals.

We had 4 model levels, with the sex - interaction (${\varvec{B}}_{3}$) being the variable of primary interest. If ${y}_{IA}$ is the CBCL score for a given individual at a given assessment age, ${\epsilon }_{IA}$ is the error term, ${B}_{0}$ is the intercept and ${u}_{I0}$ is the random effect of the participant on the intercept, our models were as follows:

1) An unadjusted model including only a fixed effect of BW (${B}_{1}$).

2) An unadjusted model as in 1) but with a fixed effect of sex (${B}_{2}$) and a sex interaction term (${\varvec{B}}_{3}*{sex}_{IA}$) where females were the reference category,

3) A model as in 2), but with confounders as detailed in the directed acyclic graph (DAG) (${B}_{cov}$) and the age at assessment (${B}_{4}$) added as fixed effects

4) A parsimonious model with main effects and interaction. The parsimonious model was derived using backward variable removal from the full model 3) by examining *p*-values from the linear mixed effects model. Only variables with a *p*-value < 0.2 were included in model 4)

The full model, including interactions and confounders (model 3 and 4) was specified as follows:

$$\begin{array}{l}{y_{IA}} = {B_0} + {u_{I0}} + ({B_1} + {B_3} * se{x_{IA}}) * B{W_{IA}}\\+ {B_2} * se{x_{IA}} + {B_4} * ag{e_{IA}} + {B_{cov}} * co{v_{IA}} + {\epsilon _{IA}}\end{array}$$

For prespecified sensitivity analyses, we excluded preterm births (less than 37 weeks gestational age at birth) to ensure robustness of results in the term cohort; additionally, aggressive behaviour was reassessed with the inclusion of an age 2 behavioural assessment using the preschool form of the CBCL. We added a fixed term to the model to account for the increased number of items on the preschool aggression problems. Finally, as parent characteristics have an association with both BW and CBCL scores, it is possible that parents of children with lower BW could rate behaviour differently and bias the estimate of childhood phenotype; therefore, we applied our parsimonious model covariables to the teacher ratings at age 10 in a linear regression with bootstrapping of standard errors. Post-hoc we examined how dichotomisation of variables at extremes of BW and CBCL-scores affected parsimonious model output. To model the dichotomised outcomes we used a generalised mixed effects model with a logit link. Graphical illustration of effects was performed with the plot_model function from the sjPlot package in R. We used the STROBE cohort checklist when writing our paper [42].

## Results

In the Raine Study, 2868 (100%) live births were assessed for eligibility (Supplementary Fig. 2). BW and sex were recorded for 2269 (79.1%) participants with a CBCL assessment from ages 5–17 (used in the model 1 + model 2 regression). Information on DAG-determined confounders was available for 1994 (69.5%) participants (used in the model 3 regression). Gestational age was dropped from the aggressive behaviour parsimonious model 4 for a total of 1996 (69.6%) participants. Maternal baseline variables were evenly distributed between pregnancies with male and female offspring (Table 1). The mean BW in the Raine Study cohort was 3318.5 g (1-SD = 594.5 g) and males had higher BW than females. Males had higher CBCL scores across the examined subscales including teacher assessments and preschool assessments (Table 2 and supplementary Table 3). CBCL follow-up decreased as the cohort age increased with 1076 participants completing all 5 assessments, a mean of 3.95 assessments and substantial attrition at age 17. Compared to dropouts, the Raine Study participants with at least 1 CBCL assessment had more favourable socio-economic status, less pregnancy-related risk behaviour and a higher BW (supplementary Table 1).

**Table 1:** Baseline demographics for the analytic cohort (*n* = 2269)

	Female (*n* = 1095)	Male (*n* = 1174)
**Birth weight (g)**		
	3251.1 (± 586.0)	3381.4 (± 595.6)
**Maternal age at birth (years)**		
Mean (SD)	28.6 (± 5.9)	28.5 (± 5.7)
Missing	0 (0%)	1 (0.1%)
**Income level (AUD)†**		
Mean (SD)	3.7 (± 1.2)	3.7 (± 1.2)
Missing	68 (6.2%)	50 (4.3%)
**Maternal body mass index (kg/m^2)**		
Mean (SD)	22.3 (± 4.3)	22.4 (± 4.2)
Missing	0 (0%)	2 (0.2%)
**Maternal ethnicity**		
European descent	972 (88.8%)	1,058 (90.1%)
Aboriginal	15 (1.4%)	16 (1.4%)
Polynesian	10 (0.9%)	8 (0.7%)
Vietnamese	2 (0.2%)	4 (0.3%)
Chinese	50 (4.6%)	49 (4.2%)
Indian	32 (2.9%)	27 (2.3%)
Other	14 (1.3%)	11 (0.9%)
Missing	0 (0.0%)	1 (0.1%)
**Maternal education††**		
Mean (SD)	1.2 (± 1.5)	1.2 (± 1.5)
Missing	0 (0%)	1 (0.1%)
**Diabetes or hypertension**		
Absent	914 (83.5%)	967 (82.4%)
Present	181 (16.5%)	206 (17.5%)
Missing	0 (0.0%)	1 (0.1%)
**Gestational age at birth (weeks)**		
Mean (SD)	38.8 (± 2.2)	38.8 (± 2.1)
Missing	1 (0.1%)	1 (0.1%)
**Smoking in pregnancy†††**		
Mean (SD)	0.7 (± 1.3)	0.5 (± 1.2)
Missing	87 (7.9%)	80 (6.8%)
**Psychiatric disorder**		
Absent	1,073 (98.0%)	1,147 (97.7%)
Present	22 (2.0%)	26 (2.2%)
Missing	0 (0.0%)	1 (0.1%)
**Maternal alcohol consumption††††**		
Mean (SD)	4.8 (± 1.4)	4.8 (± 1.3)
Missing	0 (0%)	1 (0.1%)

†Family income: 1 = Less than $7,000, 2=$7,000-$11,999, 3=$12,000-$23,999, 4=$24,000-$35,000, 5=$36,000 or more

††Education: 0 = None or `Other`, 1 = Trade certificate or apprenticeship, 2 = Professional registration (non-degree), 3 = College diploma or degree, 4 = University degree

††† 0 = None, 1 = 1 to 5 daily, 2 = 6 to 10 daily, 3 = 11 to 15 daily, 4 = 16 to 20 daily, 5 = 21 or more per day

††††1 = Daily, 2 = Several times per week, 3 = Approximately once per week, 4, Less than once per week, 5 = One binge effort, 6 = Never

**Table 2:** Child behaviour checklist (CBCL) scores for the analytic cohort (*n* = 2269)

	Female (*n* = 1095)	Male (*n* = 1174)	*P*-value
**CBCL Attention problems age 5**			**< 0.0001**
Mean (SD)	2.6 (± 2.8)	3.6 (± 3.1)	
Missing	97 (8.9%)	114 (9.7%)	
**CBCL Attention problems age 8**			**< 0.0001**
Mean (SD)	2.5 (± 2.9)	3.7 (± 3.5)	
Missing	134 (12.2%)	157 (13.4%)	
**CBCL Attention problems age 10**			**< 0.0001**
Mean (SD)	2.0 (± 2.8)	3.2 (± 3.4)	
Missing	173 (15.8%)	181 (15.4%)	
**CBCL Attention problems age 14**			**< 0.0001**
Mean (SD)	1.9 (± 2.6)	2.8 (± 3.2)	
Missing	265 (24.2%)	307 (26.1%)	
**CBCL Attention problems age 17**			**0.0007**
Mean (SD)	1.6 (± 2.4)	2.1 (± 2.7)	
Missing	443 (40.5%)	512 (43.6%)	
**CBCL Aggression problems age 5**			**< 0.0001**
Mean (SD)	7.8 (± 5.7)	9.5 (± 6.7)	
Missing	97 (8.9%)	114 (9.7%)	
**CBCL Aggression problems age 8**			**< 0.0001**
Mean (SD)	6.6 (± 5.7)	8.5 (± 6.9)	
Missing	134 (12.2%)	157 (13.4%)	
**CBCL Aggression problems age 10**			**< 0.0001**
Mean (SD)	5.4 (± 5.2)	7.1 (± 6.3)	
Missing	173 (15.8%)	181 (15.4%)	
**CBCL Aggression problems age 14**			**0.036**
Mean (SD)	5.2 (± 5.6)	5.9 (± 6.1)	
Missing	265 (24.2%)	307 (26.1%)	
**CBCL Aggression problems age 17**			**0.75**
Mean (SD)	3.8 (± 4.9)	3.9 (± 4.9)	
Missing	443 (40.5%)	512 (43.6%)	
**CBCL Social problems age 5**			**0.033**
Mean (SD)	1.6 (± 1.7)	1.8 (± 1.9)	
Missing	97 (8.9%)	114 (9.7%)	
**CBCL Social problems age 8**			**0.11**
Mean (SD)	1.6 (± 1.8)	1.8 (± 2.2)	
Missing	134 (12.2%)	157 (13.4%)	
**CBCL Social problems age 10**			**0.0003**
Mean (SD)	1.4 (± 2.0)	1.8 (± 2.2)	
Missing	173 (15.8%)	181 (15.4%)	
**CBCL Social problems age 14**			**0.077**
Mean (SD)	1.1 (± 1.8)	1.3 (± 2.0)	
Missing	265 (24.2%)	307 (26.1%)	
**CBCL Social problems age 17**			**0.93**
Mean (SD)	0.8 (± 1.5)	0.7 (± 1.4)	
Missing	443 (40.5%)	512 (43.6%)	

*P*-value calculated by Willcoxon rank-sum test

**Fig. 1:**
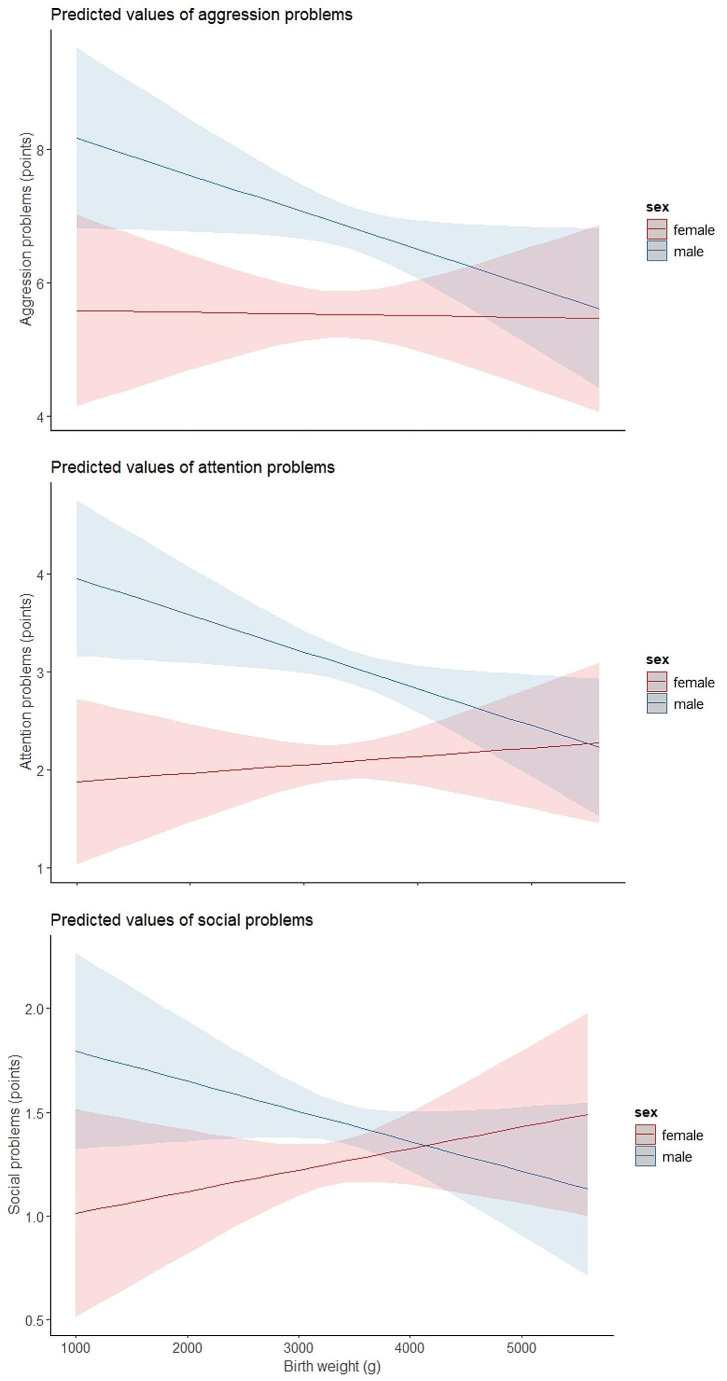
Graphical illustration of model output Graphical illustration of the estimated overall linear associations between birth weight and scores of aggression, attention and social problems measured by the Child behaviour checklist from ages 5–17 in the parsimonious confounder adjusted model. Note that the y-axis changes for each scale due to the different number of items and that the sex difference of interest (association of birthweight and behaviour) is captured in the slope of the lines, not the mean differences between the lines. The shaded area represents 95% CI

For aggressive behaviour, there was no significant main effect of BW in the unadjusted model 1(B: -0.0872, 98.3%CI: [-0.294, 0.130]) (Table 3). We found a significant sex x BW interaction in the unadjusted model 2 (B: -0.436, 98.3%CI: [-0.844, -0.0253]) with a stronger association for aggressive behaviour observed for males. The sex x BW interaction diminished after confounder adjustment in our complete (B: -0.315, 98.3%CI: [-0.744, 0.127]) model 3 and parsimonious (B: -0.310, CI: [-0.742, 0.140]) model 4 and did not show any significant interaction between BW and male sex. (Table 3; Fig. 1)

**Table 3:** The association between BW and aggression problems in the Raine Study ages 5–17

	Univariable model (Model 1)	Crude sex-interaction (Model 2)	Fully adjusted (Model 3)**	Parsimonious model (Model 4)***
**Main effect**	B: -0.0872 CI*: [-0.294, 0.130] SE: 0.089 *P*-value*: 0.33	NA	NA	NA
**Baseline BW effect (females)**	NA	B: 0.0602 CI*: [-0.191, 0.315] SE: 0.106 *P*-value*: 0.57	B: -0.00802 CI*: [-0.386, 0.380] SE: 0.161 *P*-value*: 0.96	B: -0.0160 CI*: [-0.335, 0.298 ] SE: 0.133 *P*-value*: 0.90
**Sex Interaction (male sex)**	NA	**B: -0.436** CI*: [-0.844, -0.0253] **SE: 0.172** P-value*: 0.011	B: -0.315 CI*: [-0.744, 0.127] SE: 0.182 *P*-value*: 0.08	B: -0.310 CI*: [-0.742, 0.140] SE: 0.185 *P*-value*: 0.09

* P-value is approximated based on Z-statistic of the bootstrapped SE, but significance is derived from the 98.3 % CI

** Adjusted for age at assessment, maternal BMI, maternal education,maternal psychiatric illness, gestational age at birth, maternal age at birth, maternal smoking during pregnancy, diabetes mellitus or hypertension in pregnancy, family income during pregnancy, maternal ethnicity and maternal alcohol consumption during pregnancy

*** Adjusted for age at assessment, maternal BMI, maternal age at birth, gestational age at birth, maternal smoking during pregnancy, family income level and maternal ethnicity

For attention problems, there was a significant inverse linear effect of BW in the unadjusted model 1 (B: -0.131, 98.3%CI: [-0.227, -0.0252]) with a 1 SD increase in BW reducing attention problems by 0.131 points (Table 4). We found a significant sex x BW interaction in the unadjusted model 2 (B: -0.334 98.3%CI: [-0.530, -0.137]) with male sex driving the association between BW and attention problems in the overall sample. The sex interaction was robust to multivariable adjustment in the complete model 3 (B: -0.276 98.3%CI: [-0.503, -0.0388]) and in the parsimonious model 4 (B: -0.274 98.3%CI: [-0.507, -0.0432]) (Table 4; Fig. 1).

**Table 4:** The association between BW and attention problems in the Raine Study ages 5–17

	Univariable model (Model 1)	Crude sex-interaction (Model 2)	Fully adjusted (Model 3)**	Parsimonious model (Model 4)***
**Main effect**	**B: -0.131** CI*: [-0.227, -0.0252] **SE: 0.042** P-value*: 0.002	NA	NA	NA
**Baseline BW effect (females)**	NA	B: -0.0156 CI*: [-0.138, 0.112] SE: 0.052 *P*-value*: 0.77	B: 0.0579 CI*: [-0.137, 0.255] SE: 0.082 *P*-value*: 0.48	B: 0.0509 CI*: [-0.141, 0.246] SE: 0.081 *P*-value*: 0.53
**Sex Interaction (male sex)**	NA	**B: -0.334** CI*: [-0.530, -0.137] **SE: 0.08** P-value*: < 0.0001	**B: -0.276** CI*: [-0.503, -0.0388] **SE: 0.097** P-value*:0.0045	**B: -0.274** CI*: **[-0.507, -0.0432]** **SE: 0.097** P-value*: 0.0047

* P-value is approximated based on Z-statistic of the bootstrapped SE, but significance is derived from the 98.3 % CI

** Adjusted for age at assessment, maternal BMI, maternal education,maternal psychiatric illness, gestational age at birth, maternal age at birth, maternal smoking during pregnancy, diabetes mellitus or hypertension in pregnancy, family income during pregnancy, maternal ethnicity and maternal alcohol consumption during pregnancy

*** Adjusted for age at assessment, maternal BMI, maternal age at birth, gestational age at birth, maternal smoking during pregnancy, family income level and maternal ethnicity

For social problems, there was a significant inverse linear effect of BW in the unadjusted model 1(B: -0.0646 98.3%CI: [-0.123 -0.0043]) with a 1 SD increase in BW reducing social problems by 0.0646 points (Table 5). We found a significant sex x BW interaction in the unadjusted model 2 (B: -0.164 98.3%CI: [-0.283, -0.0441]), with male sex driving the association between BW and social problems in the overall sample. The sex interaction was robust to multivariable adjustment in the complete model 3 (B: -0.149 98.3%CI: [-0.286, -0.013]) and the parsimonious model 4 (B: -0.148 98.3%CI: [-0.285, -0.00734]) (Table 5; Fig. 1).

**Table 5:** The association between BW and social problems in the Raine Study ages 5–17

	Univariable model (Model 1)	Crude sex-interaction (Model 2)	Fully adjusted (Model 3)**	Parsimonious model (Model 4)***
**Main effect**	**B: -0.0646** CI*: [-0.123 -0.0043] **SE: 0.025** P-value*: 0.0096	NA	NA	NA
**Baseline BW effect (females)**	NA	B: 0.00839 CI*: [-0.075, 0.093] SE: 0.035 *P*-value*: 0.81	B: 0.0663 CI*: [-0.0574, 0.193] SE: 0.052 *P*-value*: 0.21	B: 0.0617 CI*: [-0.0601, 0.184] SE: 0.051 *P*-value*: 0.23
**Sex Interaction (male sex)**	NA	**B: -0.164** CI*: [-0.283, -0.0441] **SE: 0.05** P-value*: 0.001	**B: -0.149** CI*: [-0.286, -0.013] **SE: 0.058** P-value*: 0.009	**B: -0.148** CI*: [-0.285, -0.00734] **SE: 0.058** P-value*: 0.011

* P-value is approximated based on Z-statistic of the bootstrapped SE, but significance is derived from the 98.3 % CI

** Adjusted for age at assessment, maternal BMI, maternal education, maternal psychiatric illness, gestational age at birth, maternal age at birth, maternal smoking during pregnancy, diabetes mellitus or hypertension in pregnancy, family income during pregnancy, maternal ethnicity and maternal alcohol consumption during pregnancy

*** Adjusted for age at assessment, maternal BMI, maternal psychiatric illness, maternal age at birth, gestational age at birth, maternal smoking during pregnancy, maternal alcohol consumption during pregnancy and family income level

For sensitivity analyses of aggressive behaviour, we had CBCL data from the age 2 round of assessments for aggressive behaviour. Inclusion yielded an additional 115 participants for regression (model 1 *n* = 2384). A full reanalysis with inclusion of age 2 assessments did not change the estimate size or significance (supplementary Table 3).

Exclusion of preterm births (*n* = 147) had little effect on the estimate parameters for social- and attention problems (supplementary Table 4). We saw a 30% reduction in the beta coefficient of the BW x sex interaction for aggressive behaviour.

Age 10 teacher assessment of child behaviour with the TRF was available for 1585 individuals with relevant covariables for the model 4 regression. No sex x BW interaction reached significance at the 98.3% level, but we saw directionally similar associations between BW and childhood assessments as was seen with the parent assessments (supplementary Table 5). Post-hoc we used a BW category of < 2500 g as the exposure. This approach yielded consistent results for attention problems, but diminished results for social problems (supplementary Table 6). When using a “borderline” cut-off for CBCL scores (T-score > = 67) estimates were drastically reduced (supplementary Table 7).

## Discussion

Using repeated parent ratings across childhood and adolescence we examined crude and confounder-adjusted sex differences in the association between BW and aggression, attention, and social problems from ages 5–17 years. We found longitudinal sex difference in the relationship between birth weight and attention problems and social problems, but not aggression problems.

We found no BW x sex interaction in aggressive behaviour and so could not reproduce the Dooley et al. findings showing a male vulnerability. Although there was a crude association in our model 2, the signal weakened with multiple regression in models 3 and 4. The inclusion of age 2 assessments, exclusion of preterm births or assessment by teachers did not change inference. Our findings also contrast with results from Momany et al. [22], who, similar to Dooley et al. found a BW x sex interaction. Both corrected for variables collected at the time of assessment. One explanation for this discrepancy could be that we did not have the power to detect a difference; however, our study sample was more than double that of Momany et al. Alternatively, it is possible that the earlier findings were subject to residual confounding. Although confounders of the sex interaction are randomly allocated (through fetal sex), perinatal risks, specifically smoking, may have sex-specific effects on BW [43]. Perinatal risks can be markers of genetic liability to poor mental health [37] and inadequate confounder adjustment could bias previous estimates. Including perinatal risks collected during pregnancy should reduce such confounding, and maternal smoking survived backwards variable removal in all parsimonious models underscoring the association with child behaviour.

We found a BW x sex interaction in attention problems, confirming a male vulnerability. This finding was robust across all models. When converting the parsimonious effect-estimates to kg, they were larger but comparable to the Dooley paper (0.46 (95% CI 0.07–0.85) vs. 0.35 (95% CI 0.19–0.51) points per kg BW) [20]. The exclusion of preterm births did not dramatically change the effect size. Sensitivity analysis using raw scores from the TRF was in directional agreement with CBCL scores. The TRF models were not significant at our Bonferroni corrected threshold; however, there was a signal when using the 95% CI (data not shown). Although not conclusive, this data supports the results from the primary analysis. Our finding is also in line with the finding from Momany et al. [22]. Our finding is inconsistent with results from Murray et al., who found a female vulnerability in the Brazilian birth cohort PELOTAS [44]. It is not clear why our results show opposite findings, but there are differences between our designs that might contribute. First, Murray et al. used a very early behaviour assessment (age 4), and males and females have been known to manifest attention problems differently across childhood and adolescence [44]. Second, they used categorical exposures (low vs. appropriate birth weight) with a 2500 g threshold for both males and females, although BW spectra differ between males and females. Third, they approached the CBCL as an ordinal outcome variable, whereas we treated it as a continuous outcome. Fourth, they had a markedly different study sample compared to us, characterised by less affluent mothers with different pregnancy-related risk behaviours, which might change the relationship between BW and parent assessment. Our results also contrast with the apparent null finding from the sibling control study by Pettersson et al. [6]. Although not compared directly, they found a similar relationship between BW and rates of neurodevelopmental disorders diagnosed across life in males and females (supplementary Table 9 in [6]). It is important to note that “neurodevelopmental disorders” also encompass autism; furthermore, a diagnostic category is different from the continuous spectrum in the CBCL attention problem scale. Recent developments have suggested that psychopathology may be better viewed as dimensional traits [45]. Our supplementary analysis using categories of CBCL suggests that this could have important implications for inference regarding sex differences. In addition, incidence of ADHD diagnosis peaks later in girls as compared to boys [46], which might suggest that the age 17 cut-off of in our data collection represents a period of symptomatic latency for females [26].

We found a BW x sex interaction in social problems suggesting a male vulnerability. This finding was robust across all models. The exclusion of preterm births did not change the inference, and teacher assessment agreed with the results from the primary analysis. There have been previous reports of an overall association between lower birth weight and social problems in childhood and adulthood [47, 48]; however, Dooley et al. were to our knowledge the first authors to examine a sex difference directly and did not find a significant sex difference. Our results suggest that lower birth weight increases social problems in males but given the novelty of this finding replication is needed. The social problems scale measures problems with peers (e.g. “Gets teased a lot”) and immaturity (e.g. “Prefers being with younger kids”) and correlates with sustained attention [49] meaning that social problems could lie downstream of a primary effect on attention problems. Social problems have also been associated with autism [50] and monozygotic twin studies suggest increased autism from lower BW [51].

The biology explaining increased male social- and attention problems at lower BW are not elucidated by the current study, but white matter vulnerability during fetal development is well-recognised [52]. Male white matter growth in temporal and frontal regions is increased during periods of rapid growth [19]. Although not stratified by sex, MRI follow-up of LBW in adulthood demonstrates a persistent loss of white matter integrity in these areas [53]. In turn, frontal and temporal structures are important determinants of social cognition [54] and attention problems [55]. Studies in rats have suggested a male cellular vulnerability of myelin-forming cells to stressors [56] and others have reported reduced placental gene expression of enzymes forming the hormonal feto-placental barrier in LBW [57]. Whether direct cellular damage or neuromodulation from maternal hormones could underly such white matter changes in lower BW is unclear.

This study’s strengths are the prospective data collection (avoiding recall bias), limited attrition, and use of multiple well-validated psychometric instruments. The models are also robust in using repeated measures on individuals and a careful approach to model construction using DAGs to make explicit the relationship between our variables. This study’s limitations are the primarily Caucasian sample in the cohort and the age 17 cut-off for psychometric evaluation; furthermore, reports of a secular trend of increasing BW (around 24 g from 1995 to 2005) could limit the generalisability of reported effect sizes in present day. Changes in parental and medical practise from the 1990s to present day aimed at ameliorating adverse behaviour postnatally could also result in an altered association between lower BW and behaviour throughout childhood and adolescence, including the sex differences in this association.

In conclusion, using repeated measures from ages 5–17 with correction of maternal baseline variables collected during pregnancy, we were able to show a male vulnerability of lower birth weight in the development of attention problems and social problems; however, we failed to find a similar sex-interaction for the development of aggressive behaviour. Future studies should be careful in selecting continuous or categorical outcomes, as potential sex-differences at the extremes may differ from those found when considering total sample behaviour; furthermore, sex differences in brain morphology associated with variation in BW and potential postnatal mediators such as parenting strategies are potential areas of future research.

## Electronic supplementary material

Below is the link to the electronic supplementary material.

Supplementary Material 1

## Data Availability

The first author of this paper had full access to all the data in the study and take responsibility for the integrity of the data and the accuracy of the data analysis.

## References

[1] Swanson JD, Wadhwa PM (2008) Developmental origins of child mental health disorders. J Child Psychol Psychiatry 49(10):1009–1019 19017021 10.1111/j.1469-7610.2008.02014.x PMC2862633

[2] Bateson P et al (2004) Developmental plasticity and human health. Nature 430(6998):419–421 15269759 10.1038/nature02725

[3] de Loret C et al (2014) Low birth weight, preterm birth and small for gestational age association with adult depression: systematic review and meta-analysis. Br J Psychiatry 205(5):340–347 25368358 10.1192/bjp.bp.113.139014

[4] Sømhovd MJ et al (2012) Anxiety in adolescents born preterm or with very low birthweight: a meta-analysis of case-control studies. Dev Med Child Neurol 54(11):988–994 22924489 10.1111/j.1469-8749.2012.04407.x

[5] Franz AP et al (2018) Attention-Deficit/Hyperactivity disorder and very Preterm/Very low Birth Weight: a Meta-analysis. Pediatrics, 141(1) 10.1542/peds.2017-1645 29255083

[6] Pettersson E et al (2019) Association of fetal growth with General and specific Mental Health conditions. JAMA Psychiatry 76(5):536–543 30725083 10.1001/jamapsychiatry.2018.4342 PMC6495458

[7] Tore EC et al (2018) The Association of Intrapair Birth-Weight Differences with Internalizing and externalizing behavior problems. Twin Res Hum Genet 21(3):253–262 29642972 10.1017/thg.2018.13

[8] Bohnert KM, Breslau N (2008) Stability of Psychiatric outcomes of Low Birth Weight: a longitudinal investigation. Arch Gen Psychiatry 65(9):1080–1086 18762594 10.1001/archpsyc.65.9.1080

[9] Ráčková L et al (2021) Birth weight rather than birth length is associated with childhood behavioural problems in a Czech ELSPAC cohort. PLoS ONE 16(7):e0253607 34324515 10.1371/journal.pone.0253607 PMC8321223

[10] Carlsson T et al (2021) Early environmental risk factors for neurodevelopmental disorders - a systematic review of twin and sibling studies. Dev Psychopathol 33(4):1448–1495 32703331 10.1017/S0954579420000620 PMC8564717

[11] Vaske J, Newsome J, Boisvert D (2013) The Mediating effects of Verbal skills in the Relationship between Low Birth Weight and Childhood Aggressive Behaviour. Infant Child Dev 22(3):235–249

[12] Murray E et al (2015) Differential effect of intrauterine growth restriction on childhood neurodevelopment: a systematic review. BJOG: Int J Obstet Gynecol 122(8):1062–1072 10.1111/1471-0528.13435 25990812

[13] van der Vlugt ER et al (2020) Sex- and growth-specific characteristics of small for gestational age infants: a prospective cohort study. Biology Sex Differences 11(1):25 10.1186/s13293-020-00300-z PMC7201715 32370773

[14] Stevenson DK et al (2000) Sex differences in outcomes of very low birthweight infants: the newborn male disadvantage. Arch Dis Child Fetal Neonatal Ed 83(3):F182–F185 11040165 10.1136/fn.83.3.F182 PMC1721180

[15] DiPietro JA, Voegtline KM (2017) The gestational foundation of sex differences in development and vulnerability. Neuroscience 342:4–20 26232714 10.1016/j.neuroscience.2015.07.068 PMC4732938

[16] Fink G et al (2018) Overall and sex-specific associations between fetal adversity and child development at Age 1 year: evidence from Brazil. Am J Epidemiol 187(11):2324–2331 29982368 10.1093/aje/kwy141 PMC6211242

[17] Hintz SR et al (2006) Gender differences in neurodevelopmental outcomes among extremely preterm, extremely-low-birthweight infants. Acta Paediatr 95(10):1239–1248 16982497 10.1080/08035250600599727

[18] Hindmarsh GJ et al (2000) Gender differences in cognitive abilities at 2 years in ELBW infants. Extremely low birth weight. Early Hum Dev 60(2):115–122 11121674 10.1016/s0378-3782(00)00105-5

[19] Studholme C, Kroenke CD, Dighe M (2020) Motion corrected MRI differentiates male and female human brain growth trajectories from mid-gestation. Nat Commun 11(1):3038 32546755 10.1038/s41467-020-16763-y PMC7297991

[20] Dooley N et al (2022) Birth Weight and Childhood Psychopathology in the ABCD Cohort: Association is Strongest for attention problems and is moderated by sex. Res Child Adolesc Psychopathol 50(5):563–575 35072847 10.1007/s10802-021-00859-0 PMC9054906

[21] Murray E et al (2015) Sex differences in the association between foetal growth and child attention at age four: specific vulnerability of girls. J Child Psychol Psychiatry 56(12):1380–1388 25879754 10.1111/jcpp.12422

[22] Momany AM et al (2017) Sex moderates the impact of birth weight on child externalizing psychopathology. J Abnorm Psychol 126(2):244–256 27868421 10.1037/abn0000238 PMC5305621

[23] Dooley N et al (2023) The persistent effects of foetal growth on child and adolescent mental health: longitudinal evidence from a large population-based cohort. Eur Child Adolesc Psychiatry 32(10):2067–2076 35861893 10.1007/s00787-022-02045-z PMC10533650

[24] Achenbach TM (1991) Manual for the child behavior Checklist/4–18 and 1991 Profile. University of Vermont, Department of Psychiatry

[25] Achenbach TM (1966) The classification of children’s psychiatric symptoms: a factor-analytic study. Psychol Monogr 80(7):1–37 5968338 10.1037/h0093906

[26] Momany AM, Kamradt JM, Nikolas MA (2018) A Meta-analysis of the Association between Birth Weight and attention deficit hyperactivity disorder. J Abnorm Child Psychol 46(7):1409–1426 29159441 10.1007/s10802-017-0371-9 PMC5962386

[27] Girard L-C (2021) Concomitant trajectories of Internalising, Externalising, and peer problems across childhood: a person-centered Approach. Res Child Adolesc Psychopathol 49(12):1551–1565 34279766 10.1007/s10802-021-00851-8 PMC8557151

[28] Robbers SC et al (2011) Trajectories of CBCL attention problems in childhood. Eur Child Adolesc Psychiatry 20(8):419–427 21713506 10.1007/s00787-011-0194-0 PMC3141842

[29] Newnham JP et al (1993) Effects of frequent ultrasound during pregnancy: a randomised controlled trial. Lancet 342(8876):887–891 8105165 10.1016/0140-6736(93)91944-h

[30] Dontje ML, Eastwood P, Straker L (2019) Western Australian pregnancy cohort (Raine) study: generation 1. BMJ Open 9(5):e026276 31138581 10.1136/bmjopen-2018-026276 PMC6549642

[31] White SW et al (2017) The Raine study had no evidence of significant perinatal selection bias after two decades of follow up: a longitudinal pregnancy cohort study. BMC Pregnancy Childbirth 17(1):207 28662683 10.1186/s12884-017-1391-8 PMC5492127

[32] Ramsay R, Kamphaus (2002) Essentials of behavioural assessment. Wiley eBook

[33] Achenbach TM, Ruffle TM (2000) The child Behavior Checklist and related forms for assessing behavioral/emotional problems and competencies. Pediatr Rev 21(8):265–271 10922023 10.1542/pir.21-8-265

[34] Ivanova MY et al (2007) Testing the 8-syndrome structure of the child behavior checklist in 30 societies. J Clin Child Adolesc Psychol 36(3):405–417 17658984 10.1080/15374410701444363

[35] Ivanova MY et al (2007) Testing the teacher’s Report Form syndromes in 20 societies. School Psychol Rev 36(3):468–483

[36] Morisaki N et al (2017) Social and anthropometric factors explaining racial/ethnical differences in birth weight in the United States. Sci Rep 7(1):46657 28429791 10.1038/srep46657 PMC5399358

[37] Havdahl A et al (2022) Associations between pregnancy-related predisposing factors for offspring neurodevelopmental conditions and parental genetic liability to Attention-Deficit/Hyperactivity disorder, Autism, and Schizophrenia: the Norwegian mother, Father and Child Cohort Study (MoBa). JAMA Psychiatry 10.1001/jamapsychiatry.2022.1728 PMC9260642 35793100

[38] Core Team R (2021) *R: A language and environment for statistical computing. R Foundation for statistical computing*

[39] Robitzsch A (2020) Why ordinal variables can (almost) always be treated as continuous variables: clarifying assumptions of robust continuous and ordinal factor analysis estimation methods. Front, Educ

[40] Schielzeth H et al (2020) Robustness of linear mixed-effects models to violations of distributional assumptions. Methods Ecol Evol 11(9):1141–1152

[41] Thai HT et al (2013) A comparison of bootstrap approaches for estimating uncertainty of parameters in linear mixed-effects models. Pharm Stat 12(3):129–140 23457061 10.1002/pst.1561

[42] Vandenbroucke JP et al (2007) Strengthening the reporting of Observational studies in Epidemiology (STROBE): explanation and elaboration. Ann Intern Med 147(8):W163–W194 17938389 10.7326/0003-4819-147-8-200710160-00010-w1

[43] Zarén B, Lindmark G, Bakketeig L (2000) Maternal smoking affects fetal growth more in the male fetus. Paediatr Perinat Epidemiol 14(2):118–126 10791654 10.1046/j.1365-3016.2000.00247.x

[44] Murray AL et al (2019) Sex differences in ADHD trajectories across childhood and adolescence. Dev Sci 22(1):e12721 30156362 10.1111/desc.12721

[45] Kotov R et al (2017) The hierarchical taxonomy of psychopathology (HiTOP): a dimensional alternative to traditional nosologies. J Abnorm Psychol 126(4):454–477 28333488 10.1037/abn0000258

[46] Dalsgaard S et al (2020) Incidence rates and cumulative incidences of the full spectrum of diagnosed Mental disorders in Childhood and Adolescence. JAMA Psychiatry 77(2):155–164 31746968 10.1001/jamapsychiatry.2019.3523 PMC6902162

[47] Mathewson KJ et al (2017) Mental health of extremely low birth weight survivors: a systematic review and meta-analysis. Psychol Bull 143(4):347–383 28191983 10.1037/bul0000091

[48] Mendonça M, Bilgin A, Wolke D (2019) Association of Preterm Birth and Low Birth Weight with romantic Partnership, sexual intercourse, and parenthood in Adulthood: a systematic review and Meta-analysis. JAMA Netw Open 2(7):e196961–e196961 31298716 10.1001/jamanetworkopen.2019.6961 PMC6628597

[49] Yung TWK et al (2021) Examining the role of attention deficits in the social problems and withdrawn behavior of children with sluggish cognitive tempo symptoms. Frontiers in Psychiatry, p 12 10.3389/fpsyt.2021.585589 PMC8129013 34017271

[50] Mazefsky CA et al (2011) Child behavior checklist scores for school-aged children with autism: preliminary evidence of patterns suggesting the need for Referral. J Psychopathol Behav Assess 33(1):31–37 22661827 10.1007/s10862-010-9198-1 PMC3362998

[51] Isaksson J et al (2023) Evaluation of Birth Weight and neurodevelopmental conditions among monozygotic and dizygotic twins. JAMA Netw Open 6(6):e2321165–e2321165 37389871 10.1001/jamanetworkopen.2023.21165 PMC10314302

[52] Tsimis ME et al (2016) Risk factors for periventricular white matter injury in very low birthweight neonates. Am J Obstet Gynecol 214(3):380 .e1-380.e6 10.1016/j.ajog.2015.09.108 26454132

[53] Eikenes L et al (2012) Being born small for gestational age reduces white matter integrity in adulthood: a prospective cohort study. Pediatr Res 72(6):649–654 23007032 10.1038/pr.2012.129

[54] Filley CM (2020) Social Cognition and White Matter: Connectivity and Cooperation. Cogn Behav Neurol, 33(1) 10.1097/WNN.0000000000000223 32132405

[55] Connaughton M et al (2022) White matter microstructure in children and adolescents with ADHD, vol 33. Clinical, NeuroImage, p 102957 10.1016/j.nicl.2022.102957 PMC8842077 35149304

[56] Yasuda K et al (2020) Sex-specific differences in transcriptomic profiles and cellular characteristics of oligodendrocyte precursor cells. Stem Cell Res 46:101866 32563975 10.1016/j.scr.2020.101866

[57] Phuthong S et al (2020) Sex differences in Placental Protein Expression and efficiency in a rat model of fetal Programming Induced by maternal undernutrition. Int J Mol Sci, 22(1) 10.3390/ijms22010237 PMC7795805 33379399

