# Supplementary material for: Sex-specific effects of birth weight on longitudinal behavioural outcomes in children and adolescents: findings from the raine study
Source: Eur Child Adolesc Psychiatry. 2024 May 9;33(12):4157–68. doi: 10.1007/s00787-024-02450-6 (PMC11618204; doi:10.1007/s00787-024-02450-6)
Supplement: Supplementary file 1 — Supplementary Material 1 [file 787_2024_2450_MOESM1_ESM.docx]

# Supplementary methods

Formulation of the questionnaire data used in adjusted models:

*During the period that you smoked, what was the average number of cigarettes per day that you smoked? 0 = None 1 = 1 – 5 daily 2 = 6 – 10 daily 3 = 11 – 15 daily 4 = 16 – 20 daily 5 = 21 or more per day*

*Which of the following would you describe yourself as?: 1 = Caucasian (European descent) 2 = Aboriginal 3 = Polynesian 4 = Vietnamese 5 = Chinese 6 = Indian 8 = Other, specify: _____________*

*What was your total family income before tax, per year, at the time you became pregnant?: 1. Less than $7,000 2. $7,000 - $11, 999 3. $12,000 - $23,999 4. $24,000 - $35,999 5. $36,000 or more 77. Family income unknown (e.g. adolescent at home)*

*Since leaving school have you completed any further education? : 0 = None 1 = Trade certificate or apprenticeship 2 = Professional registration (non-degree) e.g. Nursing, police 3 = College Diploma or Degree eg. TAFE / WAIT / WACAE 4 = University degree 5 = Other, What type of education: ________________________*

*What is your usual weight when you are not pregnant? : _____ stones _____ lbs OR _____ kg*

*The next section asks about your previous medical history. Has any doctor ever told you that you had any of the following? (0 = No, 1 = Yes) Treated Hypertension: ____ Pregnancy induced Hypertension : Diabetes: ____ Gestational diabetes: ____ Psychiatric disorder: ____ ____*

*During the first 3 months of this pregnancy would you say that you drank alcohol: 1 = Daily 2 = Several times a week 3 = Approximately once a week 4 = Less than once a week 5 = Never*

# Supplementary Figure 1 – Directed Acyclic Graph


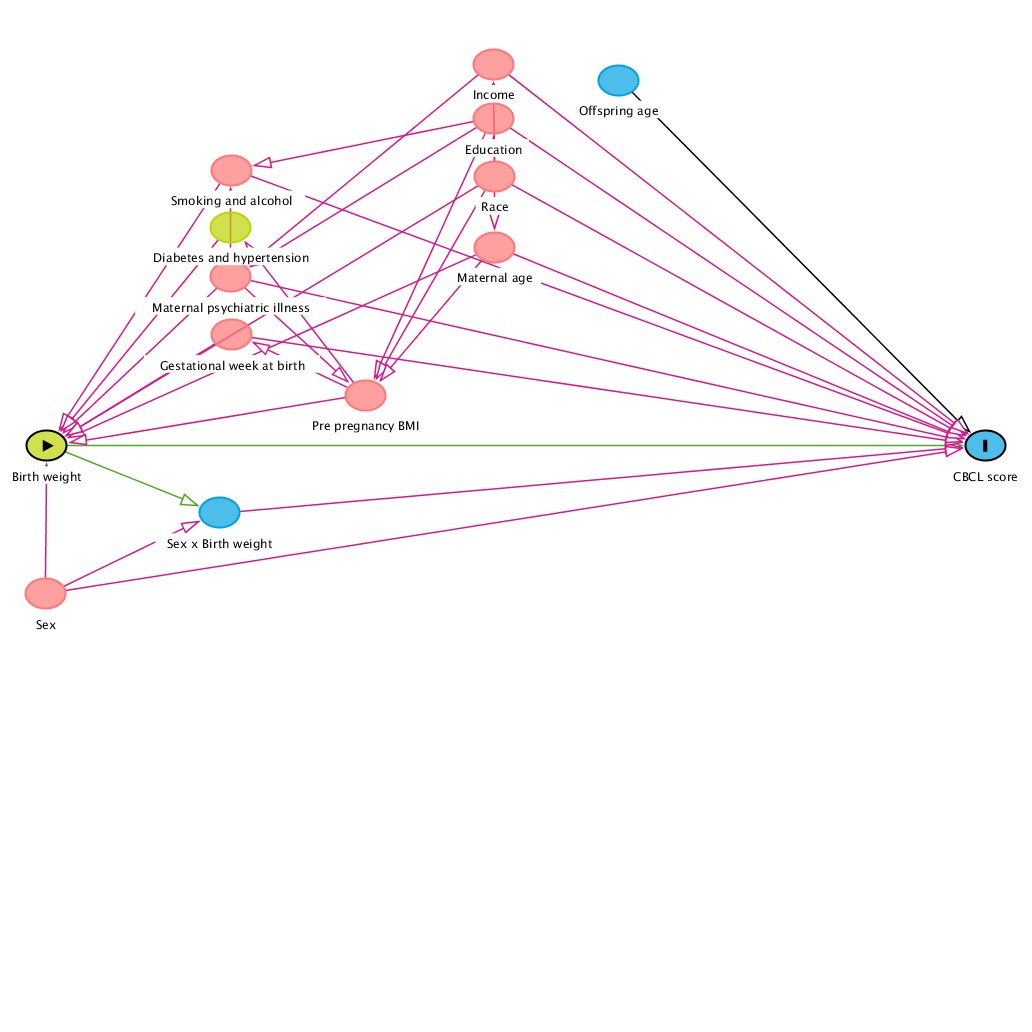


Figure 1. Conceptual framework for the primary analysis of sex-specific effects of birthweight on childhood behaviour with possible maternal and pregnancy-related confounders recorded in gestational week 18 of pregnancy. The primary variable of interest was the interaction between birthweight and sex on CBCL-scores. In order to avoid adjusting for downstream effects, prenatal variables were chosen; however, the age at assessment was included to reduced noise from increasing age. **CBCL**: Child Behaviour Checklist 4-18 years, **BMI**: Body Mass Index.

# Supplementary Figure 2 – Flowchart of cohort selection


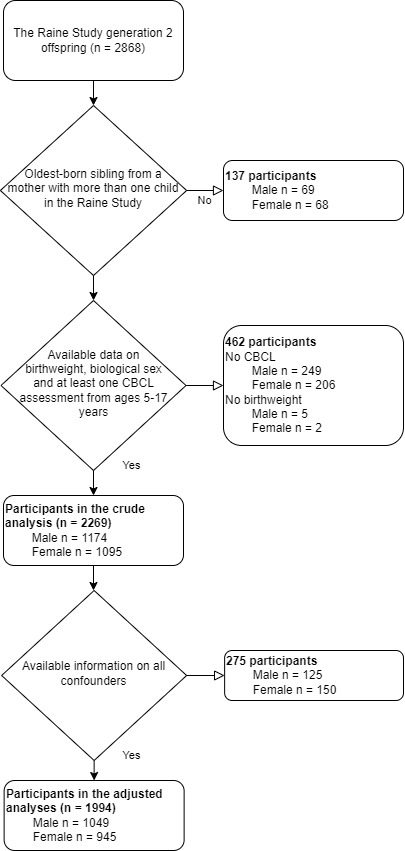


Figure 2. Flow diagram of cohort selection. **CBCL:** Child behaviour checklist 4/18 years

| Supplementary Table 1. Demographics for analytic cohort vs excluded participants (n = 2868) | | |
| --- | --- | --- |
|  | **Excluded prticipants (n=599)** | **Analytic cohort (n=2269)** |
| **Birth weight (g)** | | |
| Mean (SD) | 3159.0 (±702.2) | 3318.5 (±594.5) |
| Missing | 10 (1.7%) | 0 (0%) |
| **Maternal age at birth (years)** | | |
| Mean (SD) | 26.1 (±6.0) | 28.5 (±5.8) |
| Missing | 71 (11.9%) | 1 (0.0%) |
| **Income level (AUD)†** | | |
| Mean (SD) | 3.2 (±1.3) | 3.7 (±1.2) |
| Missing | 112 (18.7%) | 118 (5.2%) |
| **Maternal body mass index (kg/m^2)** | | |
| Mean (SD) | 22.4 (±4.6) | 22.3 (±4.2) |
| Missing | 63 (10.5%) | 2 (0.1%) |
| **Maternal ethnicity** | | |
| European descent | 442 (73.8%) | 2,030 (89.5%) |
| Aboriginal | 36 (6.0%) | 31 (1.4%) |
| Polynesian | 6 (1.0%) | 18 (0.8%) |
| Vietnamese | 2 (0.3%) | 6 (0.3%) |
| Chinese | 26 (4.3%) | 99 (4.4%) |
| Indian | 15 (2.5%) | 59 (2.6%) |
| Other | 9 (1.5%) | 25 (1.1%) |
| Missing | 63 (10.5%) | 1 (0.0%) |
| **Maternal education††** | | |
| Mean (SD) | 0.8 (±1.3) | 1.2 (±1.5) |
| Missing | 63 (10.5%) | 1 (0.0%) |
| **Diabetes or hypertension in pregnancy** | | |
| Absent | 450 (75.1%) | 1,881 (82.9%) |
| Present | 86 (14.4%) | 387 (17.1%) |
| Missing | 63 (10.5%) | 1 (0.0%) |
| **Gestational age at birth (weeks)** | | |
| Mean (SD) | 38.1 (±3.0) | 38.8 (±2.2) |
| Missing | 9 (1.5%) | 2 (0.1%) |
| **Smoking in pregnancy†††** | | |
| Mean (SD) | 1.0 (±1.6) | 0.6 (±1.2) |
| Missing | 152 (25.4%) | 167 (7.4%) |
| **Treatment for psychiatric disorder** | | |
| Absent | 518 (86.5%) | 2,220 (97.8%) |
| Present | 18 (3.0%) | 48 (2.1%) |
| Missing | 63 (10.5%) | 1 (0.0%) |
| **Maternal alcohol consumption††††** | | |
| Mean (SD) | 5.0 (±1.3) | 4.8 (±1.3) |
| Missing | 63 (10.5%) | 1 (0.0%) |
| †Family income: 1=Less than $7,000, 2=$7,000-$11,999, 3=$12,000-$23,999, 4=$24,000-$35,000, 5=$36,000 or more | | |
| ††Education: 0 = None or `Other`, 1 = Trade certificate or apprenticeship, 2=Professional registration (non-degree), 3=College diploma or degree, 4=University degree | | |
| ††† 0=None, 1=1 to 5 daily, 2=6 to 10 daily, 3=11 to 15 daily, 4=16 to 20 daily, 5=21 or more per day | | |
| ††††1=Daily, 2=Several times per week, 3=Approximately once per week, 4, Less than once per week, 5=One binge effort, 6=Never | | |

| Supplementary table 2. Additional behavioural assessments used in sensitivity analyses | | | |
| --- | --- | --- | --- |
|  | **Female (n = 1095)** | **Male (n = 1174)** | **P-value** |
| **CBCL Aggression problems age 2** | | | **0.002** |
| Mean (SD) | 17.6 (±10.0) | 19.2 (±10.6) |  |
| Missing | 248 (22.6%) | 244 (20.8%) |  |
| **TRF Aggression problems age 10** | | | **< 0.0001** |
| Mean (SD) | 2.0 (±4.4) | 5.2 (±7.6) |  |
| Missing | 251 (22.9%) | 248 (21.1%) |  |
| **TRF Attention problems age 10** | | | **< 0.0001** |
| Mean (SD) | 2.9 (±5.2) | 7.3 (±8.1) |  |
| Missing | 251 (22.9%) | 248 (21.1%) |  |
| **TRF Social problems age 10** | | | **< 0.0001** |
| Mean (SD) | 1.1 (±2.6) | 1.9 (±3.0) |  |
| Missing | 251 (22.9%) | 248 (21.1%) |  |
| P-value calculated by Willcoxon rank-sum test. **TRF: Teacher report form, CBCL: Child behaviour checklist 2-3 years** | | | |

Supplementary Table 3. The association between BW and aggressive behaviour in the Raine Study ages 2-17

* P-value is approximated based on Z-statistic of the bootstrapped SE, but significance is derived from the 98.3 % CI

** Adjusted for age at assessment, maternal BMI, maternal education, maternal psychiatric illness, gestational age at birth, maternal age at birth, maternal smoking during pregnancy, diabetes mellitus or hypertension in pregnancy, family income during pregnancy, maternal ethnicity and maternal alcohol consumption during pregnancy.

*** Adjusted for age at assessment, maternal BMI, maternal age at birth, maternal smoking during pregnancy, maternal alcohol consumption, family income level and maternal ethnicity

|  | Model 1 | Model 2 | Model 3* | Model 4* |
| --- | --- | --- | --- | --- |
| **Main effect** | B: -0.136  CI*: [-0.344, 0.0904]  SE: 0.091  P-value: 0.135 | NA | NA | NA |
| **Baseline BW effect (females)** | NA | B: 0.00885  CI*: [-0.258, 0.302]  SE: 0.117  P-value: 0.94 | B: 0.0118  CI*: [-0.404, 0.450]  SE: 0.179  P-value: 0.948 | B: -0.0795  CI*: [-0.413, 0.264]  SE: 0.142  P-value: 0.57 |
| **Sex Interaction (male sex)** | NA | B: -0.449  CI*: [-0.882, -0.0354]  SE: 0.177  P-value: 0.011 | B: -0.297  CI*: [-0.795, 0.189]  SE: 0.21  P-value: 0.149 | B: -0.279  CI*: [-0.736, 0.178]  SE: 0.191  P-value: 0.15 |

|  | Aggressive behaviour** | Attention problems*** | Social problems**** |
| --- | --- | --- | --- |
| **Baseline BW effect (females)** | B: -0.0893  CI*: [-0.471, 0.302]  SE: 0.162  P-value: 0.58 | B: 0.00266  CI*: [-0.201, 0.209]  SE: 0.086  P-value: 0.98 | B: 0.0599  CI*: [-0.0748, 0.195]  SE: 0.057  P-value:0.29 |
| **Sex Interaction (male sex)** | B: -0.220  CI*: [-0.764, 0.316]  SE: 0.226  P-value: 0.33 | B: -0.239  CI*: [-0.506, 0.0279]  SE: 0.112  P-value: 0.032 | B: -0.175  CI*: [-0.334, -0.0093]  SE: 0.068  P-value: 0.010 |

Supplementary Table 4. The association between BW and CBCL syndrome scales in the Raine Study ages 5-17 with the exclusion of preterm births using our parsimonious model 4.

* P-value is approximated based on Z-statistic of the bootstrapped SE, but significance is derived from the 98.3 % CI

** Adjusted for age at assessment, maternal BMI, maternal age at birth, maternal smoking during pregnancy, maternal alcohol consumption, family income level and maternal ethnicity

*** Adjusted for age at assessment, maternal BMI, maternal age at birth, gestational age at birth, maternal smoking during pregnancy, family income level and maternal ethnicity

**** Adjusted for age at assessment, maternal BMI, maternal psychiatric illness, maternal age at birth, gestational age at birth, maternal smoking during pregnancy, maternal alcohol consumption during pregnancy and family income level

Supplementary Table 5. The association between BW and CBCL syndrome in the Raine Study age 10 using teacher assessments with the Teacher Report Form in the model 4 regression.

|  | Aggressive behaviour ** | Attention problems*** | Social problems **** |
| --- | --- | --- | --- |
| **Baseline BW effect (females)** | B: 0.119  CI*: [-0.238, 0.482]  SE: 0.151  P-value: 0.43 | B: 0.17  CI*: [-0.471, 0.803]  SE: 0.267  P-value: 0.52 | B: 0.052  CI*: [-0.231, 0.334]  SE: 0.118  P-value: 0.66 |
| **Sex Interaction (male sex)** | B: -0.541  CI*: [-1.31, 0.218]  SE: 0.3199  P-value: 0.091 | B: -0.75  CI*: [-1.675, 0.172]  SE: 387  P-value: 0.052 | B: -0.343  CI*: [-0.720, 0.026]  SE: 0.156  P-value: 0.0252 |

* P-value is approximated based on Z-statistic of the bootstrapped SE, but significance is derived from the 98.3 % CI

** Adjusted for age at assessment, maternal BMI, maternal age at birth, maternal smoking during pregnancy, family income level and maternal ethnicity, maternal alcohol consumption during pregnancy

*** Adjusted for age at assessment, maternal BMI, maternal age at birth, gestational age at birth, maternal smoking during pregnancy, family income level, maternal psychiatric disease and maternal ethnicity

**** Adjusted for age at assessment, maternal BMI, maternal age at birth, gestational age at birth, maternal smoking during pregnancy, maternal alcohol consumption during pregnancy, family income level, maternal psychiatric disease and maternal ethnicity

Supplementary table 6: The association between low birth weight (less than 2500 g, n=169) and CBCL-scores ages 5-17 in the model 4 regression

|  | Aggressive behaviour ** | Attention problems*** | Social problems **** |
| --- | --- | --- | --- |
| **Baseline BW effect (females)** | B: -0.190  CI*: [-1.18, 0.852]  SE: 0.42490998  P-value: 0.654243 | B: -0.45  CI*: [-1.07, 0.201]  SE: 0.266669260  P-value: 0.09492 | B: -0.278  CI*: [-0.654, 0.121]  SE: 0.162335523  P-value: 0.086841 |
| **Sex Interaction (male sex)** | B: 0.766  CI*: [-1.15, 2.55]  SE: 0.77506837  P-value: 0.322691 | B: 0.97  CI*: [-0.0692, 1.94]  SE: 0.420092467  P-value: 0.021475 | B: 0.218  CI*: [-0.350, 0.754]  SE: 0.231271614  P-value: 0.346201 |

* P-value is approximated based on Z-statistic of the bootstrapped SE, but significance is derived from the 98.3 % CI

** Adjusted for age at assessment, maternal BMI, maternal age at birth, maternal smoking during pregnancy, family income level and maternal ethnicity, maternal alcohol consumption during pregnancy

*** Adjusted for age at assessment, maternal BMI, maternal age at birth, gestational age at birth, maternal smoking during pregnancy, family income level, maternal psychiatric disease and maternal ethnicity

**** Adjusted for age at assessment, maternal BMI, maternal age at birth, gestational age at birth, maternal smoking during pregnancy, maternal alcohol consumption during pregnancy, family income level, maternal psychiatric disease and maternal ethnicity

Supplementary table 7: The association between BW and CBCL borderline category (T-score >= 67) ages 5-17 in the model 4 regression

|  | Aggressive behaviour ** | Attention problems*** | Social problems **** |
| --- | --- | --- | --- |
| **Baseline BW effect (females)** | OR: 1.01  CI*: [0.57 – 1.78]  SE: 0.29038  P-value: 0.977 | OR: 0.99  CI*: [0.53 – 1.83]  SE: 0.31486  P-value: 0.962 | OR: 1.09  CI*: [0.59 – 1.99]  SE: 0.30866  P-value: 0.782 |
| **Sex Interaction (male sex)** | OR: 0.91  CI*: [0.48 – 1.73]  SE: 0.32729  P-value: 0.782 | OR: 0.95  CI*: [0.48 – 1.85]  SE: 0.34212  P-value: 0.876 | OR: 0.84  CI*: [0.42 – 1.65]  SE: 0.34845  P-value: 0.605 |

* P-value is approximated based on Z-statistic of the bootstrapped SE, but significance is derived from the 98.3 % CI

** Adjusted for age at assessment, maternal BMI, maternal age at birth, maternal smoking during pregnancy, family income level and maternal ethnicity, maternal alcohol consumption during pregnancy. n(T-score >= 67) = 347

*** Adjusted for age at assessment, maternal BMI, maternal age at birth, gestational age at birth, maternal smoking during pregnancy, family income level, maternal psychiatric disease and maternal ethnicity. n(T-score >= 67) = 340

**** Adjusted for age at assessment, maternal BMI, maternal age at birth, gestational age at birth, maternal smoking during pregnancy, maternal alcohol consumption during pregnancy, family income level, maternal psychiatric disease and maternal ethnicity. n(T-score >= 67) = 283
